# Retention of *AnAFP* Sequence Variants in *Ammopiptanthus nanus* Ex Situ Collections with Contrasting Management Histories

**DOI:** 10.3390/plants15071060

**Published:** 2026-03-30

**Authors:** Lingling Ma, Jingdian Liu, Hongbin Li, Xiyong Wang, Daoyuan Zhang, Jiancheng Wang, Wei Shi

**Affiliations:** 1 College of Life Sciences, Shihezi University, Shihezi 832003, China; malingling200@163.com (L.M.); lihbabc@163.com (H.L.); 2 State Key Laboratory of Desert and Oasis Ecology, Key Laboratory of Ecological Safety and Sustainable Development in Arid Lands, Xinjiang Institute of Ecology and Geography, Chinese Academy of Sciences, Urumqi 830011, China; ariiiiiink@gmail.com (J.L.); wangxy@ms.xjb.ac.cn (X.W.); zhangdy@ms.xjb.ac.cn (D.Z.); 3 Turpan Eremophytes Botanic Garden, Xinjiang Institute of Ecology and Geography, Chinese Academy of Sciences, Urumqi 830011, China; 4 Xinjiang Key Laboratory of Conservation and Utilization of Plant Gene Resources, Xinjiang Institute of Ecology and Geography, Chinese Academy of Sciences, Urumqi 830011, China

**Keywords:** threatened shrub, ex situ conservation, candidate gene, founder effect, rare variant retention, conservation monitoring

## Abstract

*Ammopiptanthus nanus* (Fabaceae) is a Class II nationally protected endangered evergreen shrub in China and is endemic to the arid regions of Central Asia. To assess how contrasting ex situ management histories are associated with sequence-variant retention at an ecologically relevant gene, we analyzed a 594 bp coding fragment of the antifreeze protein gene (*AnAFP*) in one wild population and two ex situ collections maintained under active versus passive management contexts. Only two variable sites were detected across 75 individuals, both represented by single-base indels near the 5′ end of the coding region. The wild population contained both rare variants, the actively managed ex situ collection retained one of them at low frequency, and the passively maintained collection was monomorphic across the analyzed fragment. Rarefaction analysis indicated that the absence of variation in the passive collection is unlikely to be explained by sample-size disparity alone at this targeted locus. Because only one locus was analyzed, these results are interpreted as locus-specific patterns rather than evidence of genome-wide diversity change. Nevertheless, the observed pattern is consistent with reduced retention of rare sequence variants in the passive ex situ collection and with the possibility that a narrow founder base, together with the absence of subsequent genetic supplementation, contributed to this outcome. These results support the view that ex situ conservation of *A. nanus* may benefit from maximizing founder representation, maintaining sufficiently large managed collections, and combining neutral marker approaches with targeted monitoring of ecologically relevant loci. Targeted loci such as *AnAFP* should, however, be regarded as complementary indicators rather than stand-alone proxies for broader genetic diversity or adaptive potential.

## 1. Introduction

*Ammopiptanthus nanus* (M. Pop.) Cheng f. (Fabaceae) is a Tertiary relict evergreen shrub endemic to the arid regions of Central Asia. In China, it persists in highly fragmented natural populations within a narrow distributional range, giving the species considerable ecological and conservation significance [1,2]. As one of the few evergreen broadleaf shrubs in desert ecosystems, *A. nanus* contributes to community stability, wind protection, sand fixation, and the mitigation of desertification processes [3]. However, ongoing habitat fragmentation, weak natural regeneration, and increasing anthropogenic disturbance have placed persistent pressure on its wild populations, and the species is currently listed as a Class II nationally protected plant in China [4]. To reduce extinction risk and preserve germplasm resources, several ex situ collections of *A. nanus* have been established in Xinjiang. Such collections can serve as important conservation safeguards, but their genetic composition may diverge from that of source wild populations if they are founded from few individuals or maintained without continued genetic management. In conservation genetics, founder effects, demographic bottlenecks, and small effective population size can promote the stochastic loss of low-frequency variants, thereby narrowing the range of standing variation retained in ex situ collections [5,6]. This issue is especially relevant for rare or threatened plants, in which the retention of uncommon variants may be important for long-term conservation value. In addition, ex situ collections maintained under different regimes may differ in census size, propagation history, and opportunities for genetic supplementation, all of which can influence the extent to which the original variation is retained over time [7]. Evaluating how contrasting ex situ management contexts are associated with the retention or loss of sequence variants therefore remains an important task in plant conservation.

Most conservation genetic assessments rely primarily on neutral markers, such as microsatellites, chloroplast DNA sequences, or genome-wide SNPs, to infer population history and broad patterns of genetic diversity [8,9,10]. These approaches are essential for understanding demographic background, but they do not necessarily indicate whether sequence variants at loci associated with ecologically relevant traits are retained to the same extent. Trait-associated loci may show patterns that differ from those inferred from neutral variation alone, particularly when they are subject to functional constraint [11]. Accordingly, targeted analyses of sequence variation at ecologically relevant genes can provide a complementary perspective on the conservation value of ex situ germplasm, provided that the scope of inference is restricted to the locus under study and not extended to genome-wide processes [12].

In *A. nanus*, cold and freeze tolerance are ecologically relevant because the species inhabits environments characterized by strong temperature fluctuations and seasonal freezing stress [13]. Antifreeze proteins (AFPs) play important roles in plant cold acclimation by interacting with ice crystals and reducing freezing injury [14]. In related taxa, AFPs have been implicated in stress tolerance, and in *A. nanus*, the antifreeze protein gene (*AnAFP*) has been reported as a cold-related functional gene whose heterologous expression enhances cold tolerance in recombinant *Escherichia coli* and transgenic plants [13,15]. Because *AnAFP* is associated with an ecologically relevant stress-response trait, sequence variation at this locus may be informative for comparing whether ex situ collections maintained under different management contexts differ in their retention of rare sequence variants.

Our previous work on *A. nanus*, based on multiple neutral and organellar marker systems, provided a broader view of genetic structure and conservation status across wild and ex situ materials [16]. The present study was not designed to replace those broader assessments or to infer genome-wide diversity, demographic history, or effective population size from a single locus. Rather, it was intended as a targeted follow-up focused on whether a candidate gene associated with an ecologically relevant stress-response trait shows contrasting patterns of sequence-variant retention across ex situ collections with different management histories. In this sense, the *AnAFP* fragment serves as a complementary targeted marker within a broader conservation-genetic framework. Accordingly, the present study focuses specifically on sequence variation within a 594 bp fragment of the *AnAFP* coding region and evaluates how variant retention differs among one wild reference population and two ex situ collections with contrasting management histories. One ex situ collection represents a relatively active management context, characterized by larger census size, repeated propagation, and occasional supplementation with wild-derived material, whereas the other represents a more passive management context, with a narrow founder base and no known subsequent genetic supplementation. This comparison allows a locus-specific assessment of sequence-variant retention while recognizing that founder history, incomplete documentation, and local environmental differences may also have contributed to the observed patterns.

Specifically, this study was designed to: (i) characterize sequence variation at the targeted *AnAFP* locus across wild and ex situ materials of *A. nanus*; (ii) compare the retention of identified consensus sequence classes among collections maintained under contrasting ex situ management histories; and (iii) discuss how such locus-specific patterns may complement broader evaluations of ex situ germplasm. We hypothesized that an ex situ collection established from a narrow founder base and maintained under a more passive management history would be more likely to lack rare sequence variants at this targeted candidate locus than a collection maintained under a more active management history.

## 2. Results

### 2.1. Sequence Variation and Targeted Locus Characterization

A 594 bp fragment of the *AnAFP* coding region was obtained for all 75 sampled individuals. After sequence curation and alignment (see Section 4), only two variable sites were detected across the full dataset. Both were single-base indels located near the 5′ end of the coding sequence (Table 1): a +C insertion at CDS position 54 (RVar1) and an A deletion at CDS position 55 (RVar2), relative to reference sequence GQ200581.1.

Reference-based in silico translation indicates that these indels would alter the reading frame relative to the reference coding sequence. However, because no transcript-level, protein-level, or other functional validation was performed in this study, these variants are interpreted conservatively here only as curated sequence variants for locus-specific comparison, rather than as confirmed functional frameshift mutations.

**Figure 1:**
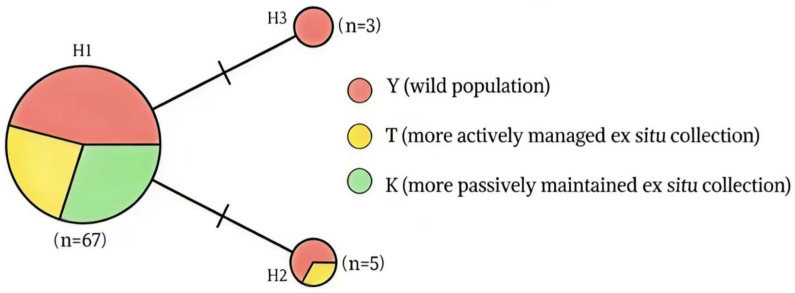
Median-joining network of curated *AnAFP* haplotypes detected across the three *A. nanus* collections. Each circle represents one haplotype (H1–H3), with circle size proportional to its total frequency in the full sample. Colors indicate the relative contribution of individuals from the wild population (Y), the relatively actively managed ex situ collection (T), and the more passively maintained ex situ collection (K). Branch hash marks indicate single mutational steps between haplotypes.

The two variants were distributed unevenly among collections. In the wild population (Y), RVar1 was detected in three individuals and RVar2 in four individuals, corresponding to three H3 consensus sequence classes and four H2 consensus sequence classes, respectively; the remaining 22 individuals carried H1. In the relatively actively managed ex situ collection (T), only RVar2 was detected, in one individual (H2), whereas the remaining 29 individuals carried H1. In the more passively maintained ex situ collection (K), no variable sites were detected, and all 16 sampled individuals carried H1 (Table 1; Figure 1).

### 2.2. Descriptive Diversity Statistics and Rarefaction Analysis

Descriptive diversity statistics for the targeted *AnAFP* fragment showed a progressive reduction in variation from the wild population to the two ex situ collections (Table 2). The wild population (Y) showed the highest variation (S = 2, h = 3, Hd = 0.197, π = 0.00044), followed by the relatively actively managed collection (T; S = 1, h = 2, Hd = 0.067, π = 0.00011). The more passively maintained collection (K) was monomorphic across the analyzed fragment (S = 0, h = 1, Hd = 0, π = 0).

**Figure 2:**
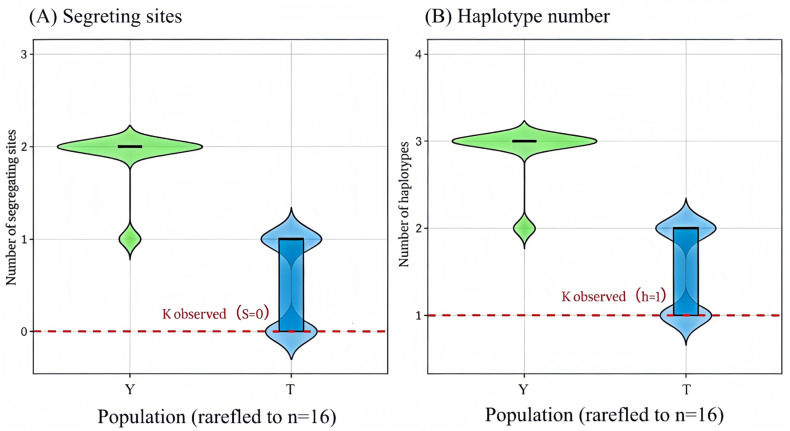
Rarefaction analysis of sequence variation at the targeted *AnAFP* locus across *Ammopiptanthus nanus* collections. (**A**) Distribution of the number of segregating sites (S) and (**B**) distribution of haplotype number (h) obtained from 1000 random subsamples without replacement after rarefying populations Y and T to 16 individuals. Green represents the wild population (Y), blue represents the more actively managed ex situ collection (T). Violin plots show the full distribution of resampled values, with embedded boxplots indicating the interquartile range and median. The red dashed line indicates the empirically observed value in K (passive collection) for each metric (S = 0 in panel A; h = 1 in panel.

To evaluate whether the monomorphism observed in K (*n* = 16) could be explained solely by its smaller sample size, rarefaction analyses were performed by randomly subsampling 16 individuals from Y and T, respectively, without replacement for 1000 replicates. In rarefied subsamples from Y, polymorphism was retained in 100% of replicates, with a mean of 1.87 segregating sites and 2.87 haplotypes per subsample; the corresponding 95% resampling intervals were 1–2 and 2–3, respectively (Figure 2; Supplementary Table S1). In rarefied subsamples from T, polymorphism was retained in 53.6% of replicates, with a mean of 0.54 segregating sites and 1.54 haplotypes per subsample; the corresponding 95% resampling intervals were 0–1 and 1–2, respectively.

By contrast, the observed K dataset remained completely monomorphic (S = 0; h = 1). Thus, even when Y and T were reduced to the same sample size as K, they still retained higher levels of sequence variation at this locus. These results indicate that the absence of variation in K is unlikely to be explained by sample-size disparity alone and is consistent with reduced retention of sequence variants at this locus.

### 2.3. Haplotype Composition and Sequence-Based Differentiation

The median-joining network resolved three haplotypes (H1–H3) separated by single mutational steps (Figure 1). H1 was the predominant haplotype, accounting for 67 of the 75 sampled individuals (Y = 22, T = 29, K = 16). H2, corresponding to the sequence carrying RVar2, was detected in five individuals in total (Y = 4, T = 1). H3, corresponding to the sequence carrying RVar1, was detected only in the wild population (Y = 3).

Sequence-based differentiation was descriptively higher between Y and K than between Y and T, whereas the T–K comparison was not informative under near-monomorphism (Table 3). Pairwise *F*_ST_ values for the targeted *AnAFP* fragment were 0.0030 between Y and T, 0.0536 between Y and K, and 0.0000 between T and K; the overall value across all three collections was 0.0235. Because K was monomorphic and T retained only one low-frequency variant, these values are presented only as descriptive locus-specific statistics and were not used to infer broader population structure, gene flow, or genome-wide differentiation.

Neutrality tests were attempted but are not interpreted because polymorphism was too low for meaningful inference; results are provided in Supplementary Table S2 for completeness only. Coding-site classification of the two curated sequence variants is provided in Supplementary Table S3.

## 3. Discussion

### 3.1. Reduced Retention of Rare AnAFP Sequence Variants in the Passive Ex Situ Collection

Using the *AnAFP* fragment as a targeted locus, this study compared the retention of sequence variants among one wild population and two ex situ collections of *A. nanus* with contrasting management histories. At this locus, the pattern was clear: the wild population (Y) contained two rare sequence variants, whereas the relatively actively managed ex situ collection (T) harbored only one of the two variants detected in the wild population, and the more passively maintained collection (K) was monomorphic. Rarefaction analysis further showed that this monomorphic pattern in K is unlikely to be explained solely by its smaller sample size.

Within the inferential limits of a single-locus study, these results are consistent with reduced retention of rare sequence variants in the passive ex situ collection. The most immediate explanation is likely founder limitation: because K appears to have been established from only a few founder plants, rare sequence variants present in the source region may not have been captured at establishment, or may have been captured only at very low frequency. Continued maintenance of a small collection without documented genetic supplementation would further reduce the likelihood that such variants would be retained in the extant collection. Recent demographic studies of *A. nanus* indicate that the species has a relatively long generation time, estimated at approximately 11 years [17]. In long-lived perennial shrubs, rare variants are generally expected to be vulnerable to stochastic loss when founder representation is narrow and maintained population size remains limited [18,19]. Although effective population size was not estimated for the collections analyzed here, the founder histories reported for both ex situ collections and the smaller maintained size of K are consistent with the general expectation that rare variants may be lost under such conditions. By contrast, the presence of one rare variant in T is consistent with the possibility that larger census size, repeated propagation, and occasional supplementation with wild-sourced material may have helped preserve at least part of the sequence variation represented in the ex situ collection.

At the same time, these comparisons should not be interpreted as direct proof that management history alone determined the observed differences. This study was retrospective, and historical records remain incomplete, especially for founder composition and establishment details. Accordingly, the most appropriate interpretation is that contrasting ex situ management histories were associated with different outcomes in *AnAFP* sequence-variant retention, rather than that a single causal factor was conclusively isolated.

### 3.2. Relationship to Broader Genetic Evidence

The locus-specific pattern observed at *AnAFP* can be discussed in relation to broader genetic evidence, but it should not be treated as a substitute for genome-wide inference. In our companion study based on multiple neutral and organellar marker systems, collection K showed stronger evidence of reduced nuclear genetic variation than the other collections, whereas organellar and ITS haplotype diversity appeared to be better preserved in K than in the other ex situ collections [16]. The complete loss of variation at *AnAFP* in K is therefore consistent with, but does not by itself demonstrate, a broader pattern of reduced nuclear variation in this collection.

This contrast between nuclear marker and organellar marker patterns may be informative for ex situ conservation. If lineage diversity is retained more strongly in organellar markers while sequence variation at a nuclear locus is lost, monitoring based only on organellar markers may overestimate the breadth of retained nuclear genetic variation. In this sense, targeted loci such as *AnAFP* may complement neutral marker approaches by indicating whether sequence variation is retained at candidate genes associated with ecologically relevant traits. However, such loci should be interpreted only as complementary indicators and not as stand-alone proxies for genome-wide diversity or adaptive potential.

### 3.3. Interpretation of the Detected Variants and Methodological Boundaries

Several methodological boundaries are important for interpreting the present results.

First, this study was based on a single targeted coding fragment of one gene. The resulting diversity estimates, haplotype network, and *F_ST_* values therefore describe only locus-specific sequence patterns and should not be extended directly to effective population size, gene flow, overall population structure, or genome-wide genetic erosion. In particular, the *F*_ST_ value of 0.0000 between T and K is not evidence that the two collections are broadly genetically similar; under conditions of monomorphism or near-monomorphism, sequence-based *F*_ST_ is only weakly informative and may obscure biologically meaningful differences [20,21].

Second, the observed indels were evaluated only through sequence curation and reference-based coding interpretation. Although in silico annotation suggests that these variants would shift the reading frame relative to the reference sequence, no transcript-level, protein-level, expression-level, or phenotypic validation was conducted. For that reason, the variants are treated here conservatively as curated sequence variants for locus-specific comparison, not as confirmed functional mutations. Alternative explanations cannot be excluded on the basis of the present data alone.

Third, *A. nanus* is reported to be diploid (2n = 18) based on previous cytogenetic studies [22,23]. Direct Sanger sequencing of PCR amplicons produced individual consensus sequences but did not resolve allelic phase. Consequently, this study did not distinguish heterozygous from homozygous states at polymorphic positions. The counts reported for the two variants therefore indicate the number of individuals carrying each variant in the consensus-sequence dataset, not genotype frequencies.

Fourth, neutrality statistics had little inferential value because polymorphism at this locus was extremely limited. The non-significant values observed for Y and T should therefore not be interpreted as evidence for neutrality, purifying selection, or demographic equilibrium [24,25].

Finally, source-related and historical confounding should be considered. Available archival information indicates that both ex situ collections originated from the Wuqia region, which reduces, but does not eliminate, the possibility that the observed difference simply reflects geographic source bias. At the same time, founder documentation remains incomplete, especially for T, and the two collections differ in post-establishment history and maintenance conditions. These factors cannot be disentangled completely in a retrospective study. However, rarefaction indicated that the lack of variation in K is unlikely to be explained by sampling effects alone. Accordingly, the most cautious interpretation is that the observed differences at this locus are consistent with the combined effects of founder representation and post-establishment history, rather than with sample-size disparity alone.

### 3.4. Cautious Implications for Ex Situ Conservation

Despite the limitations noted above, the present results suggest several cautious implications for ex situ conservation.

First, establishment from very few founder plants may fail to capture rare nuclear sequence variants, even where some broader lineage representation appears to persist. This point may be especially relevant for rare and threatened plants maintained as small legacy collections [26,27]. Second, the contrast between T and K is consistent with the view that post-establishment maintenance may affect the probability of retaining rare sequence variants. Larger maintained collection size, repeated propagation, and occasional supplementation from wild-derived material may favor the retention of rare sequence variants relative to passive maintenance without additional genetic input [6,28]. In a retrospective study, however, this interpretation should remain cautious and should not be treated as a direct test of management effectiveness. Third, monitoring strategies for ex situ germplasm may benefit from combining marker types. Neutral markers remain essential for broader demographic and diversity assessment, whereas targeted loci may provide additional information on whether rare variants at ecologically relevant genes are retained. The present study therefore suggests that loci such as *AnAFP* may be useful as complementary, rather than stand-alone, components of a multi-marker conservation framework.

## 4. Materials and Methods

### 4.1. Study Sites, Sampling Strategy, and Retrospective Study Design

**Figure 3:**
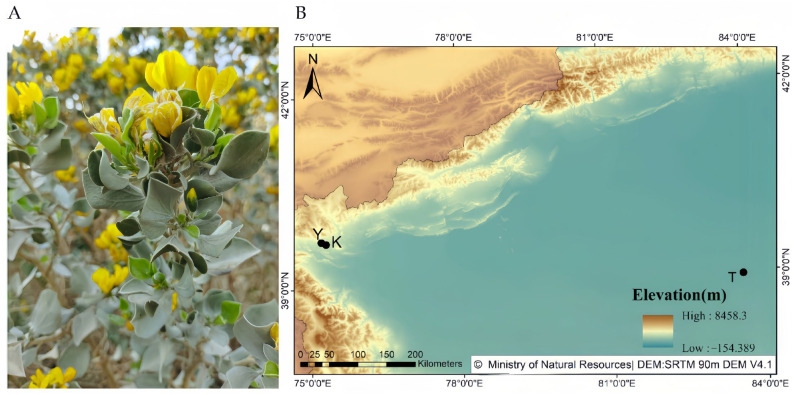
Study species and sampling locations of *Ammopiptanthus nanus.* (**A**) Flowering individual of *Ammopiptanthus nanus* from the actively managed ex situ collection at Tazhong Botanical Garden (T). (**B**) Geographic locations of the wild population (Y) and the two ex situ collections (T and K) analyzed in this study.

A total of 75 individuals of *A. nanus* were sampled in Xinjiang, China (Figure 3; Table 4), including one wild population from the Wuqia region (Y, *n* = 29) and two ex situ collections with contrasting management histories: a relatively actively managed collection at Tazhong Botanical Garden (T, *n* = 30) and a more passively maintained collection at the Forestry Academy (K, *n* = 16).

Sampling effort was adjusted to the standing size of each site. Based on field observations, collection T currently comprises a comparatively large stand (>200 mature individuals), from which 30 individuals were sampled at random. In contrast, collection K is much smaller, with an estimated 20–30 surviving individuals; thus, the 16 sampled individuals represent a high proportion of the extant collection. For the wild population Y, total census size could not be estimated reliably in the field; therefore, 29 accessible individuals were sampled across the known accessible distribution area and main accessible patches. Although the total census size of the wild population could not be estimated reliably, the sampled individuals represent the accessible portion of the local distribution and include plants from multiple accessible patches of the population. To reduce the likelihood of repeatedly sampling immediately adjacent ramets or closely neighboring individuals, sampled plants were spaced at least 10 m apart wherever possible [29]. Fresh leaves were dried immediately in silica gel and stored at −20 °C until DNA extraction.

#### Permits and Compliance

*Ammopiptanthus nanus* is a Class II nationally protected plant species in China. Field sampling and leaf collection for this study were conducted with permission from the relevant local management authorities and host institutions responsible for the study sites. All procedures complied with applicable national and regional regulations governing protected wild plant resources in China. Only a small amount of leaf material was collected from each individual, and sampling was conducted so as to minimize damage to both wild and ex situ collections.

This study was designed as a retrospective comparison of legacy ex situ collections. Historical records for both collections are incomplete. However, archival materials and curator information indicate that both ex situ collections were established from the Wuqia region, which is also the source region of the sampled wild population (Y). Available historical information further suggests that both collections were established from narrow founder bases. For K, archived records indicate seed collection from approximately 3–5 wild maternal plants. For T, curator information and archival notes likewise suggest a narrow founder base (approximately ≤5 source plants), although the exact founder composition is not formally documented. The principal contrast between T and K lies in post-establishment management: T has undergone long-term cultivation, multi-generational propagation, and occasional supplementation with wild-sourced seedlings, whereas K has received no known subsequent genetic supplementation or managed breeding and has been maintained primarily through natural regeneration.

Because this is a retrospective study, incomplete founder documentation, differences in time since establishment, and local environmental conditions cannot be fully excluded as potential confounders. Accordingly, the present comparison was intended to evaluate locus-specific patterns of sequence-variant retention across contrasting ex situ management histories, rather than to attribute all observed differences exclusively to management history alone.

### 4.2. DNA Extraction, Primer Design, PCR Amplification, and Sanger Sequencing

Genomic DNA was extracted from silica-dried leaf material using the DNAsecure Plant Kit (Tiangen, Beijing, China) according to the manufacturer’s instructions. DNA quality and concentration were checked prior to downstream analyses. To amplify the *AnAFP* locus, one pair of locus-specific primers was designed from a draft *A. nanus* genome assembly (forward: 5′-GGTATCATCAACAAGATTGGGGAG-3′; reverse: 5′-TGTATGATGTGGCAGTGGAAGATC-3′). Primer specificity was evaluated in silico using Primer-BLAST (https://www.ncbi.nlm.nih.gov/tools/primer-blast/, accessed on 20 March 2026 to reduce the likelihood of off-target amplification and to favor recovery of a single genomic target [30]. PCR amplification was performed using locus-specific primers under optimized reaction conditions for this amplicon. Products of the expected size were verified, purified, and subjected to bidirectional Sanger sequencing [31].

Because the present study focused on a single targeted sequence marker, the resulting amplicon was used for locus-specific comparison only. No additional rapidly evolving loci or genome-wide markers were generated in this study.

### 4.3. Sequence Processing and Locus-Specific Analyses

#### 4.3.1. Analytical Scope and Rationale

The *AnAFP* fragment analyzed here was treated strictly as a targeted molecular marker associated with an ecologically relevant gene. The purpose of the analysis was to compare patterns of sequence-variant retention among the wild population and the two ex situ collections. Because only a single locus was analyzed, the results were not used to infer genome-wide diversity, effective population size, gene flow, or broad demographic history. Likewise, detected nucleotide substitutions and indels were interpreted conservatively as curated sequence variants useful for locus-specific comparison, rather than as direct evidence of functional effects on protein activity or cold-tolerance phenotype.

#### 4.3.2. Sequence Assembly, Alignment, and Variant Curation

Forward and reverse Sanger reads were assembled and edited in Geneious Prime (v2024.0.5; https://www.geneious.com, accessed on 20 January 2026) [32]. Low-quality ends were trimmed, and all sequences were aligned to generate a final comparable fragment of 594 bp. Variant calling and sequence-class assignment were based only on positions retained after bidirectional sequence checking and manual curation.

Only confidently curated sequence differences were retained in the final alignment. Positions that could not be assigned unambiguously after comparison of forward and reverse reads were excluded from sequence-class definition. Insertions/deletions detected in the final alignment were treated conservatively as sequence characters contributing to sequence-class distinction. Because no transcript-level, protein-level, or independent functional validation data were generated in this study, these indels were not interpreted as confirmed functional frameshift mutations. In addition, direct Sanger sequencing of PCR amplicons did not allow allelic phasing; therefore, the present analysis does not distinguish heterozygous from homozygous states at polymorphic sites and is restricted to individual-level consensus sequences for descriptive locus-specific comparison. For ease of presentation, these curated consensus sequence classes are referred to as haplotypes throughout the manuscript, although allelic phase was not resolved.

The final curated alignment for all analyzed individuals is provided as Supplementary Data S1.

#### 4.3.3. Genetic Diversity Descriptors and Rarefaction Analysis

The number of segregating sites (S), number of haplotypes (h), haplotype diversity (Hd), and nucleotide diversity (π) were calculated in DnaSP v6.12.03 [33]. Given the single-locus design and the lack of phased allelic data, these statistics are presented only as descriptive indicators of sequence variation at the targeted *AnAFP* fragment.

To assess whether the monomorphic pattern observed in K (*n* = 16) could be explained solely by its smaller sample size relative to Y (*n* = 29) and T (*n* = 30), a rarefaction analysis was performed in R using the packages ape, pegas, and ggplot2 [34,35,36]. For Y and T, 16 sequences were randomly subsampled without replacement, matching the observed sample size of K, for 1000 replicates per population. For each replicate, we calculated the number of segregating sites (S), the number of haplotypes (h), and whether polymorphism was retained (defined as S > 0). For Y and T, summary statistics across replicates were reported as the mean, standard deviation, and 95% resampling interval for S and h, together with the proportion of replicates retaining polymorphism. The empirically observed K values (S = 0; h = 1) were then compared descriptively with the resampled distributions from Y and T. This analysis was intended to evaluate sampling sensitivity at the locus under study, rather than to estimate genome-wide diversity loss.

#### 4.3.4. Haplotype Network and Sequence Differentiation

Relationships among *AnAFP* haplotypes were reconstructed using a median-joining network in Network v10.2 [37]. Pairwise *F*_ST_ values were estimated in DnaSP [38] and are reported only as descriptive measures of sequence differentiation at this locus.

Because K was monomorphic and T was nearly monomorphic, *F*_ST_ values were interpreted with particular caution. Under such conditions, sequence-based *F*_ST_ is of limited biological informativeness for broader comparison, because the estimate is driven largely by fixation or near-fixation at one or very few sites rather than by broader allele-frequency structure [39]. Therefore, these values were not used as stand-alone evidence for gene flow, population structure, or the strength of genetic drift.

#### 4.3.5. Supplementary Neutrality Statistics

Tajima’s D and Fu and Li’s D and F statistics were calculated in DnaSP v6.12.03 [33]. However, because polymorphism at the *AnAFP* fragment was extremely limited (S ≤ 2), the statistical power of these tests was very low [24]. Accordingly, these statistics are reported only in Supplementary Table S2 for descriptive completeness and were not interpreted as evidence of selection regime or evolutionary mechanism.

## 5. Conclusions

This study provides a locus-specific analysis of sequence-variant retention at the *AnAFP* gene in *A. nanus* under contrasting ex situ management histories. At this targeted locus, the wild population retained two rare sequence variants, the relatively actively managed ex situ collection retained one of them at low frequency, and the more passively maintained collection was monomorphic. Rarefaction analysis further indicated that the absence of variation in the passive collection is unlikely to be explained by sample-size disparity alone. Within the limits of a single-locus study, these findings are consistent with reduced retention of rare *AnAFP* sequence variants in the passive ex situ collection. The observed contrast between the two ex situ collections is plausibly associated with differences in founder representation and post-establishment history, including maintained collection size and subsequent genetic supplementation, although these factors cannot be disentangled completely in a retrospective design. The present results should therefore be interpreted as a targeted-locus perspective that complements, rather than replaces, broader conservation-genetic assessments.

From a conservation perspective, the study suggests that broader founder sampling, adequately sized managed collections, and, where appropriate, periodic genetic supplementation may improve the retention of rare sequence variants in ex situ germplasm. More broadly, it illustrates the potential value of integrating neutral marker approaches with targeted monitoring of ecologically relevant candidate loci. Such loci may provide useful complementary information for conservation monitoring, but they should not be used alone to infer overall genetic diversity or adaptive potential.

## Figures and Tables

**Table 1:** High-confidence sequence variants detected in the targeted *AnAFP* fragment across the three *Ammopiptanthus nanus* collections.

Site ID	CDS Pos. (bp)	Mutation Type	Ref./Var. Allele	Reference-Based Coding Consequence	Y (Wild), *n* = 29	T (Active), *n* = 30	K (Passive), *n* = 16
RVar1	54	Indel (+1 bp)	-/C	Frameshift	3	0	0
RVar2	55	Indel (−1 bp)	A/-	Frameshift	4	1	0

Note: Variant positions are given as CDS coordinates relative to reference sequence GQ200581.1. The coding consequences shown are reference-based in silico annotations only. In the absence of transcript-level, protein-level, or independent functional validation, these variants are interpreted in this study only as curated sequence variants for locus-specific comparison. Counts refer to individuals carrying the variant in the consensus sequence derived from direct Sanger amplicon sequencing and do not distinguish heterozygous from homozygous states (see Section 4).

**Table 2:** Descriptive diversity statistics for the targeted *AnAFP* fragment in the three *A. nanus* collections.

Population	Sample Size (*n*)	Segregating Sites (S)	Haplotypes (h)	Haplotype Diversity (Hd)	Nucleotide Diversity (π)
Y (Wild)	29	2	3	0.197	0.00044
T (Active)	30	1	2	0.067	0.00011
K (Passive)	16	0	1	0.000	0.00000

Note: Statistics were calculated in DnaSP v6.12.03, treating indels as sequence polymorphisms. Values are based on individual consensus sequences from direct amplicon sequencing and do not resolve allelic phase. Because only one targeted locus was analyzed, these values are presented as descriptive indicators of locus-specific variation rather than estimates of genome-wide diversity.

**Table 3:** Pairwise and overall sequence-based *F*_ST_ estimates for the targeted *AnAFP* fragment among the three *A. nanus* collections.

Comparison	*F*_ST_ Value
Y vs. T	0.0030
Y vs. K	0.0536
T vs. K	0.0000
Overall (Y, T, K)	0.0235

Note: *F*_ST_ values were estimated in DnaSP using Hudson’s sequence-based estimator. Because K was monomorphic and T retained only one low-frequency variant, these values should be interpreted only as descriptive statistics for this locus. In particular, the T–K comparison is not biologically informative under near-monomorphism and does not imply broader genetic similarity between the two ex situ collections.

**Table 4:** Summary of the wild population and ex situ collections of *Ammopiptanthus nanus* included in this study.

Pop	Sample Size	Longitude	Latitude	Altitude (m)	Establishment History and Management Regime
Wild (Y)	29	75°10.5′ E	39°45.0′ N	2297	Natural wild population with no human intervention.
Active (T)	30	83°39.6′ E	38°58.2′ N	1100	Established ~22 years ago; founded from a limited seed collection from a small number of wild maternal source plants (exact number undocumented); large census size; intensive, multi-generational propagation with managed breeding; intermittent genetic supplementation with wild-sourced seedlings.
Passive (K)	16	75°16.2′ E	39°43.2′ N	2180	Established ~8 years ago; founded from a limited seed collection from 3–5 wild maternal source plants; small census size; no subsequent managed breeding or genetic supplementation; maintained primarily via natural regeneration.

Note: Summary of the wild population and ex situ collections of *Ammopiptanthus nanus* included in this study. Historical founder records are incomplete, especially for collection T. However, archival materials and curator information indicate that both ex situ collections originated from the Wuqia region and appear to have been established from similarly narrow founder bases. The main contrast between T and K lies in post-establishment management: T experienced long-term cultivation and occasional genetic supplementation, whereas K did not receive subsequent managed breeding or documented genetic input.

## Data Availability

The curated consensus sequence alignment generated in this study is provided in Supplementary Data S1. Additional information supporting the findings of this study is available from the corresponding author upon reasonable request.

## Supplementary Materials

The following supporting information can be downloaded at: https://www.mdpi.com/article/10.3390/plants15071060/s1, Data S1: Multiple sequence alignment (FASTA format) of the curated 594 bp *AnAFP* coding fragment for the reference sequence (GQ200581.1) and all 75 sampled individuals from the three *Ammopiptanthus nanus* collections (Y, T, and K). Table S1: Rarefaction analysis of sequence variation at the targeted *AnAFP* locus after rarefying populations Y and T to 16 individuals, with comparison to the observed values in K. Table S2: Neutrality test statistics (Tajima’s D, Fu and Li’s D, and Fu and Li’s F) for the *AnAFP* locus. Table S3: Synonymous and nonsynonymous polymorphism in the *AnAFP* gene of three *A. nanus* populations. Figure S1: Schematic representation of the sequence alignment highlighting the positions of the two indel variants (RVar1 and RVar2) relative to the reference sequence.

## Abbreviations

The following abbreviations are used in this manuscript:

*AnAFP*	*Ammopiptanthus nanus* antifreeze protein gene
CDS	Coding DNA sequence
*F*_ST_	Fixation index
Hd	haplotype diversity
Indel	Insertion/deletion
Ne	Effective population size
SNP	Single nucleotide polymorphism
π	Nucleotide diversity
