# Supplementary material for: Retention of AnAFP Sequence Variants in Ammopiptanthus nanus Ex Situ Collections with Contrasting Management Histories
Source: Plants (Basel). 2026 Mar 30;15(7):1060. doi: 10.3390/plants15071060 (PMC13074717; doi:10.3390/plants15071060)

Supplementary Materials:

Data S1: Multiple sequence alignment (FASTA format) of the curated 594 bp *AnAFP* coding fragment for the reference sequence (GQ200581.1) and all 75 sampled individuals from the three *Ammopiptanthus nanus* collections (Y, T, and K). Table S1: Rarefaction analysis of sequence variation at the targeted *AnAFP* locus after rarefying populations Y and T to 16 individuals, with comparison to the observed values in K. Table S2: Neutrality test statistics (Tajima's D, Fu and Li's D, and Fu and Li's F) for the *AnAFP* locus. Table S3: Synonymous and nonsynonymous polymorphism in the *AnAFP* gene of three *A. nanus* populations. Figure S1: Schematic representation of the sequence alignment highlighting the positions of the two indel variants (RVar1 and RVar2) relative to the reference sequence.

1. The multiple sequence alignment in FASTA format is provided as a separate file (Data S1.fasta).

2. Table S1: Rarefaction analysis of sequence variation at the targeted *AnAFP* locus after rarefying populations Y and T to 16 individuals, with comparison to the observed values in K.

Collection	original-n	rarefied-n	Replicates retaining polymorphism (%)	Mean (S)	SD (S)	95% CI lower (S)	95% CI upper (S)	Mean (h)	SD (H)	95% CI lower (h)	95% CI upper (h)
Y (Wild)	29	16	100	1.873	0.33	1	2	2.873	0.33	2	3
T (more Active)	30	16	53.6	0.536	0.498	0	1	1.536	0.498	1	2
K (more Passive)	16	16	0	0	NA	NA	NA	1	NA	NA	NA

Note: Replicates retaining polymorphism (%) indicates the proportion of rarefaction replicates with S > 0. Statistics for Y and T were calculated from 1000 subsampling replicates without replacement. NA indicates not applicable (rarefaction was not performed for the observed K dataset).

3. Table S2: Neutrality test statistics (Tajima's D, Fu and Li's D, and Fu and Li's F) for the AnAFP locus.

Population	Test Statistic	Value (p-value)
Y (Wild)	Tajima‘s D	–1.009 ($p > 0.10$)
	Fu and Li‘s D*	–0.726 ($p > 0.10$)
	Fu and Li‘s F*	–0.930 ($p > 0.10$)
T (Active)	Tajima‘s D	–1.147 ($p > 0.10$)
	Fu and Li‘s D*	–1.682 ($p > 0.10$)
	Fu and Li‘s F*	–1.766 ($p > 0.10$)
K (Passive)	Tajima‘s D	NA
	Fu and Li‘s D*	NA
	Fu and Li‘s F*	NA

Note: P-values were estimated by coalescent simulation (10,000 replicates). NA indicates no segregating sites. The tests have low power given the few segregating sites ($S \leq 2$).

4. Table S3: Synonymous and nonsynonymous polymorphism in the *AnAFP* gene of three *A. nanus* populations.

**Population**	**CDS region (bp)**	**Codons analyzed**	**Synonymous polymorphism (mS; πS)**	**Nonsynonymous polymorphism (mN; πN; θN)**	**Pairwise Ka > 0 (count/total, %)**	**Pairwise Ks**
Y (Wild)	1–594	196	0; 0.00000	1; 0.00041; 0.00054	78/406 (19.21%)	0
T (Active)	1–594	197	0; 0.00000	1; 0.00014; 0.00053	29/435 (6.67%)	0
K (Passive)	1–594	197	0; 0.00000	0; 0.00000; 0.00000	0/120 (0.00%)	0

Note: mS/mN: synonymous/nonsynonymous variable sites; πS/πN: diversity at synonymous/nonsynonymous sites; θN: Watterson's estimator for nonsynonymous sites. With πS = Ks = 0, reliable selection inference (e.g., via πN/πS or Ka/Ks) is not possible from this single locus.

5. Figure S1: Schematic representation of the sequence alignment highlighting the positions of the two indel variants (RVar1 and RVar2) relative to the reference

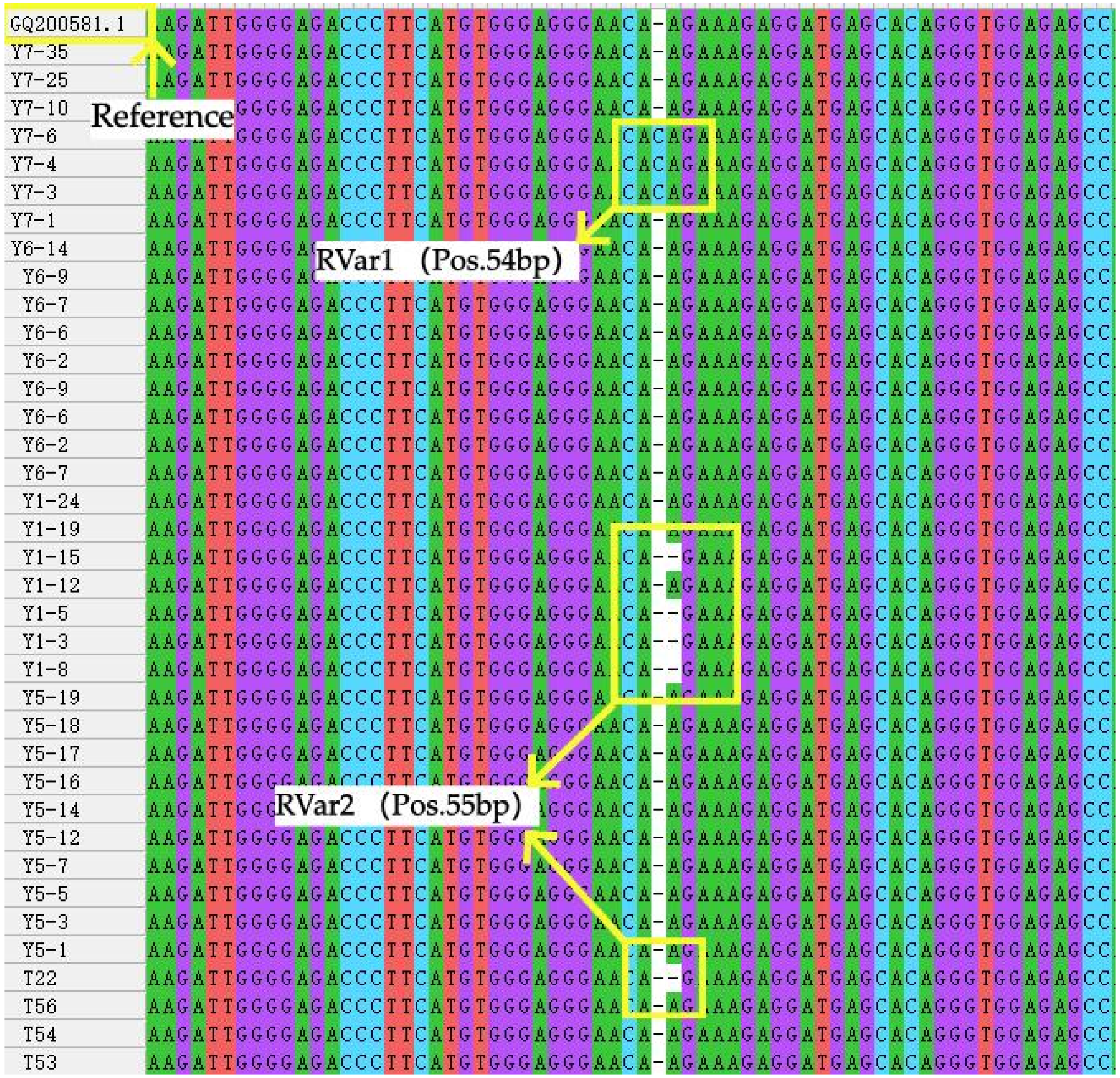

Supplement: Supplementary file 1 [file plants-15-01060-s001.zip › plants-4152425-Supplementary Materials.pdf]
